# Integrating respiratory-gated PET-based target volume delineation in liver SBRT planning, a pilot study

**DOI:** 10.1186/1748-717X-9-127

**Published:** 2014-06-02

**Authors:** Olivier Riou, Benjamin Serrano, David Azria, Benoit Paulmier, Remy Villeneuve, Pascal Fenoglietto, Antonella Artenie, Cécile Ortholan, Marc Faraggi, Juliette Thariat

**Affiliations:** 1 Radiotherapy Department, Montpellier Cancer Institute - Val d’Aurelle, 208 rue des Apothicaires, F-34298 Montpellier Cedex 5, France; 2 Physics Department, Centre Hospitalier Princesse Grace, Monaco, Monaco; 3 Nuclear Medicine Department, Centre Hospitalier Princesse Grace, Monaco, Monaco; 4 Radiotherapy Department, Centre Hospitalier Princesse Grace, Monaco, Monaco; 5 Radiotherapy Department, Centre Antoine Lacassagne, 06200 Nice, France

**Keywords:** Respiratory-gated PET, Liver metastases, SBRT, Radiotherapy planning, 4D PET

## Abstract

**Background:**

To assess the feasibility and benefit of integrating four-dimensional (4D) Positron Emission Tomography (PET) – computed tomography (CT) for liver stereotactic body radiation therapy (SBRT) planning.

**Methods:**

8 patients with 14 metastases were accrued in the study. They all underwent a non-gated PET and a 4D PET centered on the liver. The same CT scan was used for attenuation correction, registration, and considered the planning CT for SBRT planning. Six PET phases were reconstructed for each 4D PET. By applying an individualized threshold to the 4D PET, a Biological Internal Target Volume (BITV) was generated for each lesion. A gated Planning Target Volume (PTVg) was created by adding 3 mm to account for set-up margins. This volume was compared to a manual Planning Target Volume (PTV) delineated with the help of a semi-automatic Biological Target Volume (BTV) obtained from the non-gated exam. A 5 mm radial and a 10 mm craniocaudal margins were applied to account for tumor motion and set-up margins to create the PTV.

**Results:**

One undiagnosed liver metastasis was discovered thanks to the 4D PET. The semi-automatic BTV were significantly smaller than the BITV (p = 0.0031). However, after applying adapted margins, 4D PET allowed a statistically significant decrease in the PTVg as compared to the PTV (p = 0.0052).

**Conclusions:**

In comparison to non-gated PET, 4D PET may better define the respiratory movements of liver targets and improve SBRT planning for liver metastases. Furthermore, non respiratory-gated PET exams can both misdiagnose liver metastases and underestimate the real internal target volumes.

## Background

Although surgery is the standard of care for resectable liver metastases, less invasive local options like radiofrequency ablation and stereotactic body radiation therapy (SBRT) are available with promising results [1-4]. Limitations for radiation use in liver tumors (primaries or metastases) have been two-fold historically, namely limited tumor dose shaping possibilities using tridimensional irradiation due to respiratory motion and liver radiosensitivity leading in rare occasions to lethal radiation-induced liver disease (RILD) [1,5]. Technological advances in Radiation Oncology have dramatically changed the management of liver tumors, with gating and tracking on one hand and SBRT and intensity modulated radiotherapy (IMRT) techniques on the other, both allowing more conformal irradiation, and reducing RILD to less than 5% [6-8]. Treatment planning for liver radiotherapy is challenging when using computed tomography (CT) scan alone. Contrast medium injection helps to define target volumes but is insufficiently standardized and efficient to reduce inter-individual variability in delineation [9]. Magnetic Resonance Imaging (MRI) and Positron Emission Tomography (PET) are helpful to counteract this variability, but fusion uncertainty, mostly due to different respiratory cycle phases, results in unreliable volume definition when planning high precision treatment such as IMRT or SBRT [10]. Irradiation of liver tumors can be proposed to unresectable metastases and hepatocarcinomas, or patients awaiting liver grafts. Unlike for hepatocarcinoma [11], [^18^ F]FluoroDeoxyGlucose ([18 F]FDG) PET-CT imaging sensitivity to diagnose liver metastases is rather high [12].

The use of PET-CT for radiotherapy planning results in a change in target volume definition for most of the cases (84%), with an overall CTV increase of about 25% [13]. By contrast, four-dimensional (4D) PET-CT has been shown to decrease target volumes in lung cancer by as much as 34%, along with a rise in uptake values, which theoretically leads to a better tumor definition [14]. The usefulness of 4D PET-CT for radiotherapy planning has been recently evaluated in lung tumors [15,16] and for liver radiotherapy planning [10], but this study represents the first intent to compare a planning method with PET-CT to another incorporating 4D PET.

## Methods

### Liver metastases segmentation description

We investigated the reliability of establishing a personalized threshold segmentation method based on normal liver SUVmax for tumor measurements. We first evaluated this method on phantom studies and then applied the same methodology to patient cases.

This methodology is described below.

### Imaging information

The PET reconstruction parameters were as following: 2 iterations, 21 subsets, Gaussian filter (FWHM = 2 mm), 2 mm slice thickness, matrix 512×512, True X (Ordered-Subsets Expectation Maximization algorithm, Point-Spread Function with Time-Of-Flight) (Ultra HD-PET).

### Liver metastases segmentation evaluation on phantom

Phantom studies were performed with 150 seconds PET acquisition. The CT scan was used for attenuation correction and registration for PET acquisitions. We used the list mode of a Siemens Biograph mCT to get the six phase of the gated PET. The Siemens Biograph mCT was fitted with four rings and time of flight, corresponding to 32 448 LSO detectors, with each detector dimension being 4 mm × 4 mm × 20 mm. The transaxial field of view (FOV) was 50 cm and the length of the axial direction was 21.6 cm, allowing covering the whole phantom in one step.

**Figure 1:**
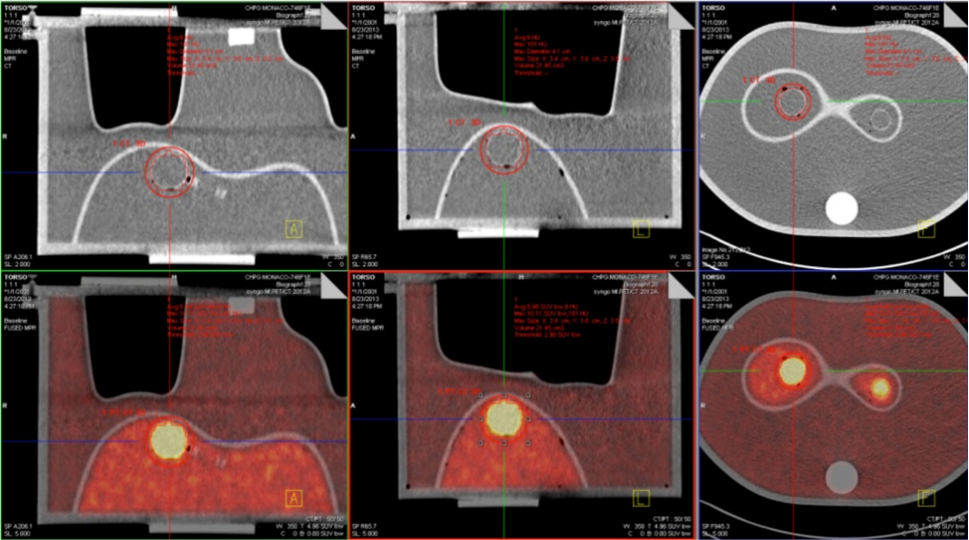
Anthropomorphic phantom Torso experiments: 3-dimensional CT and PET/CT views showing accurate volume determination on PET compared to CT (reference values for diameters) using the healthy liver background SUVmax segmentation method.

First, hot spheres were imaged in a Jaszczak phantom. The following sphere volumes (inner volume) were used: 16, 8 and 4 cc. Each sphere was successively filled to obtain the following SUV: 2, 4 and 8, in corresponding SUV backgrounds of 1.17, 1.19, and 1.23.Then phantom measurements were performed on a torso-shaped phantom, which is a semi anthropomorphic phantom more similar in shape to the patient anatomical condition: several compartments representing the liver, lungs, mediastinum, vertebras and spheres inside the liver representing the liver metastases (Figure 1). Measurements were made on a 16 cc sphere with a SUV of 7.9 and a background SUV of 2.97 and then on an 8 cc sphere with a SUV of 5.6 and a background SUV of 2.97.

The background SUVmax was used as the segmentation threshold to provide the measured volume in each experimental condition. Standardized Uptake Value (SUV) max was worked out for each experimental condition on the liver-like part of the phantom within a 20 to 30 cm^3^ ROI inside a healthy part of the liver. To have a more reliable value, 3 ROI were used when differences of less than 0.1 in the SUVmax values were obtained. Two additional measurements were added when differences between 0.1 and 0.2 were obtained. No differences higher than 0.2 were obtained.

### Patient selection

Patients with liver oligometastases eligible to SBRT treatment were consecutively included in the study.

### PET-CT and 4D PET-CT

All patients underwent a standard non-gated PET (150 seconds acquisition per bed position) and a respiratory-gated PET immediately afterwards. For a whole body non-gated PET with a mean of 7 bed positions, the acquisition was 17.5 min. The respiratory-gated PET was done during one bed position and took approximately 10 min.

The same CT scan was used for attenuation correction and registration for both PET acquisitions. CT and PET acquisition were made during uncoached quiet free breathing. PET data were acquired 60 minutes post injection of [18 F]FDG, using the list mode of a Siemens CT fitted with four rings and time of flight. The activity injected to the patients was 3.5 (±0.2) MBq/kg. The transaxial field of view (FOV) was 50 cm and the length of the axial direction was 21.6 cm, allowing covering the whole liver in one step.

The respiratory gating system consisted in an abdominal belt with a pressure sensor (AZ-733 V, Anzai Medical Co, Tokyo, Japan). The pressure signal was recorded and used for synchronized retrospective reconstructions. Six PET phases distributed equally over the breathing cycle were reconstructed for each respiratory-gated PET scan data set.

CT images were acquired by using the following parameters: pitch: 1; rotation time: 0.8 s; reconstruction: slice thickness of 2 mm and increment of 1 mm. The voltage was 120 kV and the current-time reference was 120 mAs.

The liver metastases were assessed in dimension on the different PET acquisitions with the diagnostic software from Siemens (True D) by creating Regions Of Interest (ROI) around the lesions, with an appropriate Standardized Uptake Value (SUV) threshold.

### Target volume definition

**Figure 2:**
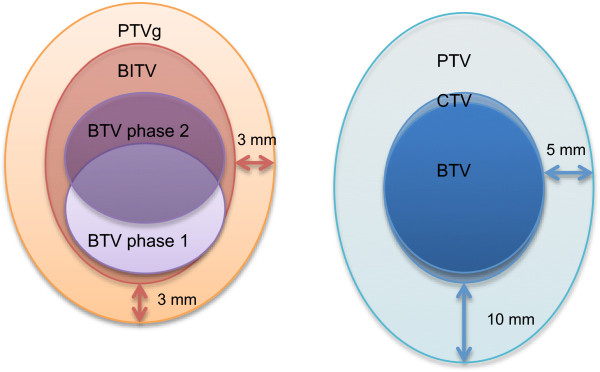
**Definition of target volumes and corresponding margins.** BTV: Biological Target Volume, semi-automatically determined on non-gated 150 second PET exam. CTV: clinical target volume, manually modified from the BTV to make it more accurate. PTV: manual Planning Target Volume, obtained by adding a 5 mm radial and a 10 mm craniocaudal margin. BTV phase 1: representation of the volume obtained on one phase (phase 1) of the 4D-PET. BTV phase 2: representation of the volume obtained on one phase (phase 2) of the 4D PET. BITV: Biological Internal Target Volume, volume obtained by merging the tumor volume on the six phases of the 4D PET. PTVg: gated Planning Target Volume, obtained by adding an isotropic 3 mm-margin to the BITV.

Target volumes were delineated using the software Mimvista version 5.2 (MIM Software Inc, Cleveland, USA). All volume measurements were made with Mimvista. The CT scan associated with the respiratory-gated PET exam was used as the planning CT scan. All the volumes are summarized on Figure 2.

For liver metastases segmentation and volume comparison, the same methodology was used between gated and non-gated PET exams in order to prevent any bias in tumor volume measurement.

Biological Target Volumes (BTV) were semi-automatically generated on the gated PET.

Normal liver Standardized Uptake Value (SUV) max was worked out for each patient within a 20 to 30 cm^3^ ROI inside a healthy part of the liver. To have a more reliable value, 3 ROI were used when differences of less than 0.1 in the SUVmax values were obtained. Two additional measurements were added when differences between 0.1 and 0.2 were obtained. No differences higher than 0.2 were obtained. These values were determined on the respiratory-gated phase of the PET exam identical to the respiratory phase of the CT scan of reference. This phase was chosen to avoid spatial errors in the determination of the ROI because of respiratory tumor motion. The threshold used to obtain the gated BTV was thus individualized for each patient as this normal liver Standardized Uptake Value (SUV) max. We used SUV normalized to body weight. This method was chosen to give the best differential value between tumor and normal liver (a SUV value above the SUV max of the normal liver was thus considered as part of the tumor). By applying the threshold to the six gated phases of the respiratory-gated PET exam and by adding these volumes, a Biological Internal Target Volume (BITV) was generated for each lesion of every patient. An isotropic 3 mm margin was added to account for set-up margins (patient positioning). The resulting volume was called gated Planning Target Volume (PTVg).

This 3 mm margin was chosen in accordance with the study of Hawkins et al., which demonstrated, by a kV cone-beam CT-based study, population random setup errors of 2.7 mm, 2.3 mm, and 3.0 mm respectively in the craniocaudal (CC), medio-lateral (ML) and antero-posterior (AP) directions [17]. Another study by Dawson et al. evaluated population random setup errors to be 2.5 mm (CC), 2.8 mm (ML), and 2.9 mm (AP) [18]. Smaller set-up errors less than 2 mm have been reported by other authors, making an isotropic 3 mm margin probably sufficient when a proper repositioning and online IGRT system is provided [19].

Images from the non-gated PET acquisition were used to help manual target volume definition. Furthermore, a non-gated BTV was semi-automatically created from the non-gated PET exam to help with manual target volume delineation on planning CT fused with non-gated PET, using the same method as for the respiratory-gated PET: normal liver SUV max was worked out for each patient within a 20 to 30 cm^3^ ROI inside a healthy part of the liver. To have a more reliable value, 3 ROI were used when differences of less than 0.1 in the SUVmax values were obtained. Two additional measurements were added when differences between 0.1 and 0.2 were obtained. No differences higher than 0.2 were obtained. The threshold used to obtain the non-gated BTV was thus individualized for each patient as this normal liver body weighted-SUV max. CT scan with contrast and/or MRI with contrast were available for target volume definition. A radiation oncologist experienced with liver SBRT treatment manually worked out the volumes, to make them more accurate, taking into account the CT scan and MRI, to better represent the real tumoral volume and excluding bones, lung and other organs-at-risk (OAR) included in the BTV. The resulting volume was called clinical target volume (CTV). An additional margin was applied to account for tumor motion and set-up margins to create a manual Planning Target Volume (PTV). A 5 mm radial and a 10 mm craniocaudal margin were chosen in accordance to the majority of the published studies on liver metastases SBRT, and considered the minimal margin to account for target volume motion and set-up margins during liver SBRT, even when active breathing control is used [6-8].

### Statistical analysis

Target volumes evaluation was based on the volumes calculated by the treatment planning system. PTV and PTVg were compared for each lesion, as well as BTV with BITV. Non-parametric Wilcoxon matched pair tests were used for comparison between PTV and PTVg, and between BTV and BITV. A two-tailed p value <0.05 was used to indicate statistical significance. All analyses were made with Instat (Graphpad Software, La Jolla, CA, USA).

## Results

### Segmentation assessment on phantoms

Differences between measured and real volumes varied from -42% to 137% using the Jaszczak phantom. Differences between measured and real volumes were 34% and 12% corresponding to mean measured volumes of 21.45 cm for the 16 cc sphere and 8.5 cm for the 8 cc sphere respectively, using the Torso phantom. Figure 1 shows 3-dimensional CT and PET/CT views and volume determination using the healthy liver background SUVmax segmentation method.

### Patient characteristics

A total of 8 patients were prospectively included in the study between December 2011 and April 2012. The total number of liver lesions evaluated was 14. Patient characteristics are shown in Table 1. The maximal diameter of the lesions was between 2 and 4.8 cm.

**Table 1:** Patient characteristics

***Patient number***	***Age***	***Gender***	***Primary cancer***	***Liver metastases***	***Maximal diameter of the lesion (cm)***	***SUV threshold (gated PET)***	***SUV threshold (non gated PET)***
1	75	F	Ampulloma	3	3.2	4.3	3.8
2	76	M	Colon	4	4.8	4.6	4
3	78	M	Lung	1	2	5	5
4	65	M	Esophageal	2	5	4.9	3.7
5	61	F	Lung	3	3.5	4.1	3
6	65	M	Colon	2	2	4.3	3.4
7	64	F	Ovarian	11	2.5	4.8	3.8
8	67	M	Lung		2.7	3.9	3.7

### SUV

One interesting result is that SUV threshold was higher with the respiratory-gated exams than with the non-gated ones. Median SUV threshold in respiratory-gated exams and non-gated exams were respectively 4.45 (min: 3.9 – max: 5.0) and 3.8 (min: 3.0 – max: 5.0). The two-tailed P value was 0.0156, considered significant.

### Metastases diagnosis

**Figure 3:**
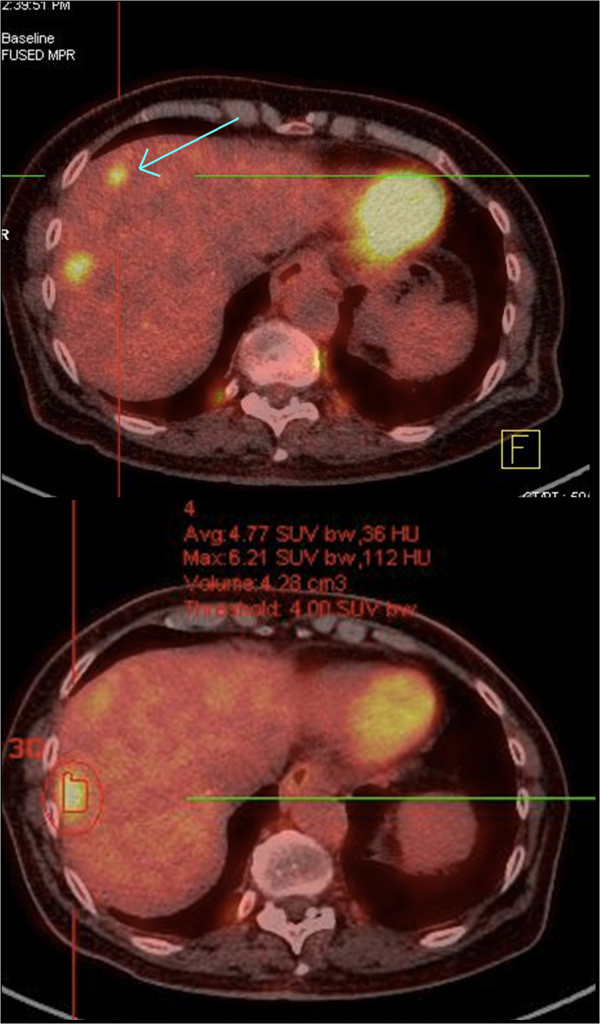
**Undiagnosed liver metastasis discovered thanks to the 4D PET exam (Upper part of the figure).** This second metastasis was not visible on the non respiratory-gated PET exam (lower part of the figure).

One undiagnosed liver metastasis was discovered thanks to the 4D PET-CT exam for patient 1 (Figure 3). This metastasis was located in segment 7 of the liver and was not visible on non-gated PET or on CT scan with contrast.

### Target volumes definition

Table 2 shows PTV and PTVg, BTV and BITV comparisons for each lesion and every patient.

**Table 2:** Volume characteristics and results for each lesion and every patient

***Patient number***	***Lesion number***	***PTV (cc)***	***PTVg (cc)***	***PTV/PTVg (%)***	***BITV (cc)***	***BTV (cc)***	***BITV/BTV (%)***	***CTV (cc)***	***Whole liver volume (cc)***
1	1	30.4	24.7	123	9.9	5	198	5.4	1671
	2	9.6	6.3	152	1.7	0.7	243	0.8	1671
2	1	29.8	41.8	71	21.6	9.4	230	5.6	1485
	2	34.6	26.4	131	12.6	7.9	159	7.9	1485
3	1	12	5.8	207	1.4	0.7	200	1.1	1530
4	1	113	73.2	154	39	34.2	114	42	1293
	2	52.8	34.4	153	17.3	14.9	116	14.6	1293
5	1	49.1	24.8	198	11.6	13.5	86	13.5	1758
	2	23.2	12.4	187	4.7	4.5	104	4.5	1758
	3	25.1	14.1	178	5.4	5	108	5	1758
6	1	20	10	200	3.5	3.2	109	3.4	2230
	2	16.4	8.5	193	2.7	1.8	150	2.2	2230
7	1	29.3	13.6	215	5.4	4.8	113	5.3	1502
8	1	16.3	12.1	134	4.4	2.4	183	2.4	1206
Mean (+ - SD)		33 (+/-26.2)	22 (+/-18.3)	150	10.1 (+/-10.3)	7.7 (+/-8.8)	131		
P value		PTV Vs PTVg	0.0052		BITV Vs BTV	0.0031			

PTV, PTVg, BTV and BITV are compared using non-parametric Wilcoxon paired tests. SD: standard deviation.

**Figure 4:**
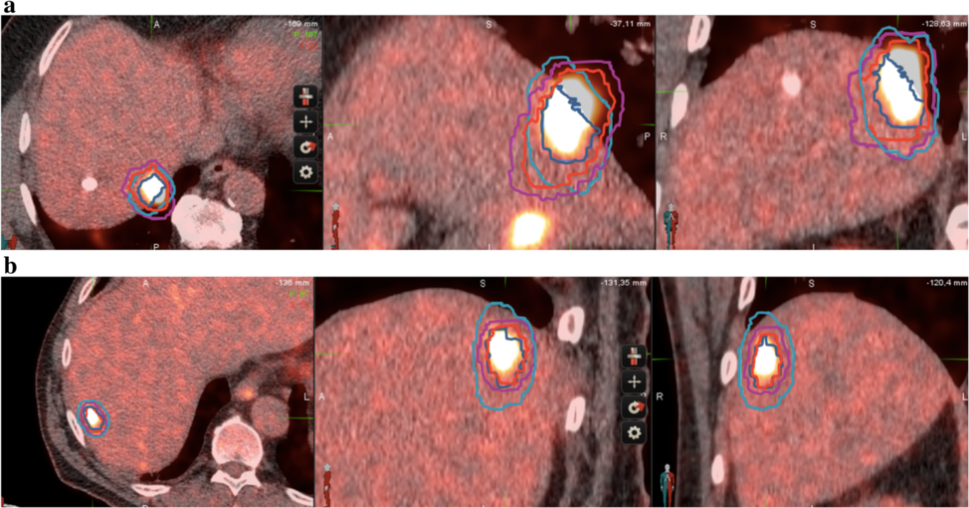
**Examples of target volume delineation. a**: Different target volumes obtained for a liver metastasis next to the diaphragm. The manual contouring leads to inaccurate evaluation of the PTV. CT scan in the upper part, non respiratory-gated PET in the middle, and co-registrated non respiratory-gated PET-CT in the lower part. CTV appears in dark blue, PTV in light blue, BITV in red and PTVg in magenta. **b**: Representative target volumes obtained for a liver metastasis (patient 6). The manual contouring leads to an increase in the PTV as compared to the PTVg. CT scan in the upper part, non respiratory-gated PET in the middle, and co-registrated non respiratory-gated PET-CT in the lower part. CTV appears in dark blue, PTV in light blue, BITV in red and PTVg in magenta.

Semi-automatically generated BTV from the non-gated PET exams were significantly lower than the BITV obtained from the respiratory gated PET: mean volume in cc (minimal and maximal values in brackets) 7.7 (0.7-34.2) Vs 10.1 (1.4-39) respectively for BTV and BITV (p = 0.0031).The use of the respiratory-gated PET exams and the resulting BITV allowed a statistically significant decrease in the Planning Target Volumes (PTV) (p = 0.0052). Mean PTVg was 22 (5.8-73.2) and mean PTV was 33 (9.6-113). PTVg were smaller than PTV in all cases but one. This exception corresponded to a metastasis adjacent to the lungs (Figure 4a). A representative example of the other cases is given on Figure 4b.

## Discussion

SBRT for liver metastases is able to precisely target the lesions while minimizing normal tissue toxicity. Dose escalation is limited by the tolerance of the surrounding healthy liver tissue. Initial local control rates are very good at 1 year but are then limited to 57 to 92% at 2 years, depending on the studies [1].

PET/CT imaging with [18 F]FDG is a well-established imaging technique with clinical implications in oncology for staging, re-staging and monitoring response to therapy, including after SBRT [20]. New software developments have highlighted its value for target volume definition and contouring purposes. However, its usefulness for radiotherapy planning has not been widely assessed and suffers from image degradation due to target movements and lack of SUV threshold standardization. As breath-holding is difficult to implement during PET/CT imaging due to the duration of the procedure, images are affected by motion artifacts and loss of contrast because of radioactivity smearing around the moving lesion, especially when the tumor is adjacent to the lungs.

Especially, cardiac and respiratory motion affects imaging because of organ displacements of up to 2 cm during normal breathing [21,22]. Liver targets are particularly exposed to respiratory motion, making a 5 mm radial and a 10 mm craniocaudal margins probably inaccurate.

Since liver SBRT is a very precise technique in a tissue exposed to radiation toxicity, any process that aims at making radiotherapy planning more precise is of particular interest. 4D PET-CT techniques are new tools to try to compensate the motion-induced image quality degradation. It has been evaluated for lung tumors [14], and has proven its clinical utility for lung tumor radiotherapy planning [15].

In this study, we checked whether 4D PET-CT was a reliable and feasible procedure for liver radiotherapy treatment planning in order to be routinely integrated to the management of SBRT patients. The use of PET-based segmentation and volumes is often complex and it is not clear how to accurately select the threshold value for segmentation [23]. Given the lack of standardized methods for liver segmentation, we developed a threshold-based method to segment metastases with respect to background SUVmax measured on patient healthy liver. The liver segmentation method was thus individualized in a timely and personalized fashion.

In lung cancer, 4D PET has been shown to better match tumor motion than non-gated PET images [24,25]. Unlike for lung tumors, no standard method has been described for automatic liver tumor segmentation using PET-CT [26-28]. The volume of the BTV mostly depends on the segmentation method used and most of these methods result in unsatisfactory volumes [29]. In head and neck tumors, it has been shown that PET-based tumor delineation yields more accurate and reproducible results with signal to background ratio than with manual/visual delineation (sensitive to window level settings and operator dependent), isocontouring based on a Standardized Uptake Value (SUV) of 2.5, or fixed percentage of the maximal SUV value (40% or 50%) delineation [30]. The signal to background method has been validated on histology for head and neck cancer and its threshold is adapted to an individual patient [31]. Its feasibility for liver malignancies was shown experimentally, with this method reliably estimating hepatic tumor diameter. The feasibility was also shown with physiological FDG liver uptake as the background value and a personalized threshold segmentation method based on normal liver SUVmax for tumor measurements. Although the SUVmax might not be the best value and peak and mean SUV might suffer fewer fluctuations, the SUVmax is widely-used in clinical practice for its convenience, as reported in the clinics and supported by our study. Additional studies evaluating these values for segmentation will hopefully improve our methodology and compare these methods to supposedly more reliable ones.

4D PET may have a potential for increased sensitivity compared to non-gated PET exam and contrast-enhanced CT scan as suggested by the discovery of an additional undiagnosed lesion on both modalities in one patient. Limitations of non-gated PET/CT include respiratory motion blur, poor spatial resolution and/or partial volume effect. Of clinical relevance, this lesion would have remained untreated without the use of 4D PET.

Moreover, respiratory-gated PET/CT was able to significantly decrease PTV as compared to a standard planning procedure using non-respiratory gated PET/CT. Furthermore, as BTV was significantly lower than BITV, automatic contouring based on non respiratory-gated exams may underestimate the real internal target volumes (that is the target volume accounting for respiratory motion) because of image blurring and inaccurate SUV values.

Improving SBRT treatment planning could therefore lead to a better protection of critical organs and further dose escalation, both possibly providing increased benefit-risk ratios.

Our study has a number of limitations. First of all, a potential drawback is that planning CT scan was made during non-gated acquisition, which implied matching a selected PET phase with the CT.

Furthermore, planning contrast-enhanced CT scan and planning MRI were not used in our study for radiotherapy planning [8,9]. However, we limited this bias by integrating PET-based imaging and semi-automatic PET-based BTV directly registered with the planning CT scan of reference that didn’t require additional hazardous image co-registration or fusion [32-34]. Further investigations will be necessary to assess the role of a respiratory-gated PET in addition to contrast-enhanced CT scan as the standard planning procedure.

## Conclusions

In comparison to non-gated PET, 4D PET may better account for the respiratory movements of liver targets and more appropriately evaluate internal target volumes. Further, reduction of irradiated liver volumes may allow for safer and potentially more efficient SBRT treatment. Finally, it might become a valuable tool to improve SBRT planning for liver metastases.

## Competing interests

The authors declare that they have no competing interest.

## Authors’ contributions

OR, BS and JT conceived the study. OR and BS collected data. OR drafted the manuscript. OR, BS, JT, PF, RV, AA, BP, MF, CO and DA participated in coordination and helped to draft the manuscript. All authors have read and approved the final manuscript.
